# Intratumor Heterogeneity of MYO18A and FBXW7 Variants Impact the Clinical Outcome of Stage III Colorectal Cancer

**DOI:** 10.3389/fonc.2020.588557

**Published:** 2020-10-29

**Authors:** Peng-Chan Lin, Yu-Min Yeh, Bo-Wen Lin, Shao-Chieh Lin, Ren-Hao Chan, Po-Chuan Chen, Meng-Ru Shen

**Affiliations:** ^1^ Department of Oncology, National Cheng Kung University Hospital, College of Medicine, National Cheng Kung University, Tainan, Taiwan; ^2^ Department of Internal Medicine, National Cheng Kung University Hospital, College of Medicine, National Cheng Kung University, Tainan, Taiwan; ^3^ Department of Computer Science and Information Engineering, College of Electrical Engineering and Computer Science, National Cheng Kung University, Tainan, Taiwan; ^4^ Department of Surgery, National Cheng Kung University Hospital, College of Medicine, National Cheng Kung University, Tainan, Taiwan; ^5^ Graduate Institute of Clinical Medicine, National Cheng Kung University Hospital, College of Medicine, National Cheng Kung University, Tainan, Taiwan; ^6^ Department of Obstetrics and Gynecology, National Cheng Kung University Hospital, College of Medicine, National Cheng Kung University, Tainan, Taiwan; ^7^ Department of Pharmacology, National Cheng Kung University Hospital, College of Medicine, National Cheng Kung University, Tainan, Taiwan

**Keywords:** colorectal cancer, whole-genome sequencing, targeted gene sequencing, tumor evolution, intratumor heterogeneity

## Abstract

Many studies failed to demonstrate benefit from the addition of targeted agents to current standard adjuvant FOLFOX chemotherapy in stage III colorectal cancer (CRC) patients. Intratumor heterogeneity may foster the resistant subclones and leads to cancer recurrence. Here, we built a cancer evolution model and applied machine learning analysis to identify potential therapeutic targets. Among 78 CRC cases, whole-genome (WGS) and deep targeted sequencing data generated from paired blood and primary tumor were used for phylogenetic tree reconstruction. Genetic alterations in the PI3K/AKT, and RTK oncogenic signaling pathways were commonly detected in founding clones. The dominant subclones frequently exhibited dysregulations in the TP53, FBXW7/NOTCH1 tumor suppression, and DNA repair pathways. Fourteen genetic mutations were simultaneously selected by random forest and LASSO methods. The logistic regression model had better accuracy (79%), precision (70%), and recall (65%) and area under the curve (AUC) (82%) for cancer recurrence prediction. Three genes, including MYO18A in the founding clone, FBXW7, and ATM in the dominant subclone, affected the prognosis were selected simultaneously by different feature sets. The *in vitro* studies, HCT-116 cells transfected with MYO18A siRNA demonstrated a significant reduction in cell migration activity by 20–40%. These results indicate that MYO18A plays a crucial role in the migration of human CRC cells. The cancer evolution model revealed the critical mutations in the founding and dominant subclones. They can be used to predict clinical outcomes and the development of novel therapeutic targets for stage III CRC.

## Introduction

Colorectal cancer (CRC) is the most commonly diagnosed gastrointestinal cancer and is also one of the leading causes of cancer-related death worldwide (1). Although adjuvant FOLFOX (5-fluorouracil, leucovorin, and oxaliplatin) chemotherapy benefits stage III CRC patients, recurrence develops in 30–35% of patients (2). Many studies have tried to assess the addition of targeted therapy, including bevacizumab and cetuximab, to FOLFOX in the adjuvant treatment of stage III CRC. However, no significant improvement in survival was noted. A considerable challenge of recurrent stage III CRC is identifying the critical genetic mutations responsible for tumor metastasis and delivering effective therapeutic strategies (3, 4). CRC is a highly heterogeneous disease that differs in clinical presentations, molecular characteristics, and responses to treatment and survival. Intratumor heterogeneity is defined as the distinct morphological and phenotypic differences within a tumor (5). Hence, building the genome evolution model underlying the mechanism of tumor carcinogenesis and biological pathways and identifying genetic markers to predict cancer recurrence is crucial to accelerate and facilitate the development of CRC treatment targets.

Cancer cells accumulate somatic alterations over time. Most cancers arise from a single clone with acquired genetic variability, and tumor progression and metastasis result from the sequential selection of more aggressive subclones (6). Cancer evolves dynamically as clonal expansions. Recent genomic studies have demonstrated that cancer relapse or metastasis is associated with the addition of new mutations and clonal evolution (7). Intratumor heterogeneity may foster tumor evolution and adaptation and hinder the biomarker development of personalized-medicine strategies that depend on results from single tumor-biopsy samples (7). The most common technology used for the molecular characterization of tumor heterogeneity is the high-throughput DNA sequencing of bulk samples. There is a significant acceleration in the use of next-generating sequencing (NGS) to approach tumor heterogeneity and evolution for precision medicine (8, 9). By using advances in bioinformatics and artificial intelligence, determining the essence of key genetic mutations in cancer evolution has recently become possible. From the evolutionary models, we can identify the “oncogenic addiction or driver” mutations that provide a fitness advantage to cancer targets against neutral “passenger” mutations.

In this study, we aimed to develop a genome evolution model by analyzing tumor heterogeneity and discovering actionable mutational targets. We first developed a cancer evolution model for the development of new agents in tumor heterogeneity and the generation of novel and more effective therapies by analyzing somatic mutations and tumor heterogeneity. Second, we established a model predicting cancer recurrence and survival and identified therapeutic driver mutation targets *via* robust optimization in machine learning. Finally, we used the causal inference model and biological methods to validate the potential cancer evolution targets. The results further described early mutation changes that predict tumors progress to stage III carcinomas and showed that statistical inference predicts that the subclone-related pathogenic mutations are acquired when the cancer is progressing. Here, we defined a broad time window of opportunity for early detection to prevent recurrence and death in advanced colorectal cancer patients. A fine-resolution view of this clonal architecture provides insight into tumor heterogeneity, evolution, and treatment response, all of which may have clinical implications.

## Materials and Methods

### Study Population

A total of 78 CRC cancer patients were recruited for the study from National Cheng Kung University Hospital (NCKUH) between January 2014 and January 2019. All CRC patients were pathological stage III and received standard surgical resection followed by adjuvant chemotherapy with the regimen of mFOLFOX6 (5-fluorouracil, leucovorin, and oxaliplatin). Clinical information was obtained from medical records. Tumor tissues and blood samples were collected at the time of enrollment. This study was approved by the Institutional Review Board of NCKUH (A-ER-103-395 and A-ER-104-153) and conducted under the Declaration of Helsinki. All participants provided written informed consent.

### Germline Whole-Genome Sequencing

Whole blood was collected for genomic DNA extraction. Genomic DNA was quantified with a Qubit fluorescence assay (Thermo Fisher Scientific) and sheared with an S2 instrument (Covaris). Library preparation was carried out using the TruSeq DNA PCR-Free HT Kit (Illumina). Individual DNA libraries were measured by 2100 Bioanalyzer (Agilent) qPCR and Qubit (Thermo Fisher Scientific). Normalized DNA libraries were combined into five-sample pools per flow cell in all eight lanes and clustered on a cBot instrument (Illumina) with Paired-End Cluster Kit V4 (Illumina). All flow cells were sequenced on the HiSeq2500 sequencer (Illumina) using the SBS Kit V4 chemistry (Illumina). FastQC was used to check read quality, and the resulting reads were aligned to the hg19 reference genome with the BWA-MEM algorithm (10). Single nucleotide variants (SNVs) and indel identification and genotyping were performed across all samples simultaneously using standard hard filtering parameters or variant quality score recalibration according to GATK Best Practices recommendations. WGS was presented with a minimum, median coverage of 30X.

### Targeted Tumor Sequencing by Cancer Panel

A total of 78 formalin-fixed paraffin-embedded primary tumor samples were collected for histologic assessment followed by the extraction of nucleic acids. The histologic evaluation was performed by pathologists, who determined the percentage of tumors and adequacy for sequencing. Tumor deep targeted sequencing was performed by Oncomine Comprehensive Assays (OCA) version 1 (Thermo Fisher Scientific) (11). OCA v1 was designed to detect 143 drug targets, including 73 hotspot genes, 49 focal copy number variation (CNV) gains, 26 genes for full coding region sequencing (CDS), and 22 fusion driver genes. (druggable) The Ion PGM Sequencing 200 Kit v.2 was used with the Ion PGM sequencer (Thermo Fisher Scientific) according to the manufacturer’s instructions. All samples were analyzed using the Torrent Suite Software 5.0.4, aligning all reads to the hg19 reference genome, and variant calling was performed running the Torrent Variant Caller plugin version 5.0.4.0. We used the ANNOVAR tool to annotate variants and filter out indels not reported in the 1000 Genomes Project, the Single Nucleotide Polymorphism Database (dbSNP), and the Exome Aggregation Consortium (ExAc) (12).

### Cancer Evolution Model Construction

The somatic mutation calling was performed by comparing the sequencing data generated by OCA v1 and germline genetic variants by WGS. Somatic SNVs were obtained by DeepSNV (13). DeepSNV (a beta-binomial model and a likelihood ratio test) is a tool that can detect subclonal SNVs with frequencies higher than 10^-4^ with higher sensitivity and specificity. The tumor subclones were identified, and clusters were identified using SciClone (14), a Bayesian clustering method. ClonEvol was used to establish the evolution tree in cancer (15).

### Statistical Analysis

Chi-square tests, Fisher’s exact tests, and unpaired t-tests were used to assess the differences between groups. Kaplan–Meier curves were used to evaluate disease-free survival, and the log-rank test was used to compare the differences between groups. Disease-free survival was defined as the time between surgery and recurrence of cancer. A P value < 0.05 was considered statistically significant.

### Pathway Analysis

Signaling pathways for frequently mutated genes detected in founding clones and dominant subclones were enriched by using Reactome (http://www.reactome.org) (16). Significance was derived from over-representation analysis built in Reactome.

### Machine Learning Analysis

#### Feature Selection

The machine learning model includes logistic regression (LR), least absolute shrinkage and selection operator (LASSO) method, and random forest. The LASSO method is a regression model that penalizes the absolute size of the coefficients, causing some regression coefficients to shrink to zero. The penalization, or constraint, allows the LASSO method to estimate a model while simultaneously performing automatic variable selection (17). The random forest (RF) model consists of an ensemble of classification trees, where each classifier was built from different independent and identically distributed bootstrap samples from a training set. Each classifier casts a vote for the most popular class. Odds ratio (OD) measures the strength of the association between two types, and hazard ratio (HR) is the ratio of the hazard rates corresponding to the conditions described by two levels of an explanatory variable in survival analysis. LASSO method was done by R package glmnet, Random forest was done by R package randomForest, the odds ratio was done by R package fmsb, and the hazard ratio was done by R package survival and survminer.

### Classifier Model

The support vector machine (SVM) (18) is a state-of-the-art classification method referred to as black-box processes. Random forest (RF) (19) is an “off-the-shelf” widely used machine learning method that shows competitive prediction performance. XGBoost (20) is an optimized implementation of gradient boosting (GBM). The advantages of the classifier include less prone to overfitting due to the strong inner regularization scheme, easy to implement parallelization and scalability. C5.0 is a machine learning method based on decision trees, which is also referred to as white box processes and is known for interpretability (21). Logistic regression is used to describe data and to explain the relationship between one dependent binary variable and one or more nominal, ordinal, interval, or ratio-level independent variables (22, 23). Finally, we performed ten-fold cross-validation on our dataset to evaluate the efficiency of the models using the caret packages in R with default parameters (24). SVM, XGBoost, and C5.0 were done by R package e1071, xgboost, and C50.

### Migration Assay

For the migration assay, placed on a cell culture surface, the ibidi Culture-Insert 2 Well (ibidi GmbH, Planegg, Germany) provides two cell cultureservoirs, each separated by a 500 μm wall. Cells were plated at 80,000 cells per well and allowed to attach overnight. On the following day, culture inserts were removed, and light microscopy images were acquired. Cells were maintained under standard culture conditions while migrating toward the cell-free gap area. For HCT-116, HT-29, and DLD-1 cells, images were acquired every 24 hours later. Images were analyzed using ImageJ software.

## Results

### Identification of Cancer Driver Mutations by Conventional Approaches

The discovery of somatic mutations that drive cancer progression is essential for therapeutic strategies. Following the protocol shown in **Supplementary Figure 1A**, we used conventional statistical methods such as odds ratio (OD) and hazard ratio (HR) to evaluate the clinical impact of somatic mutations in a cohort of 78 stage III CRC patients. The median follow-up duration of this cohort was 31.2 months. Among these patients, 33% (26/78) had recurrent disease, and 67% (52/78) remained disease-free. Of all patients, the distribution of gender was the same. The median age of these patients was 58 years old. The prevalent primary tumor site was left colon (80.8%). There was no significant difference between recurrence and tumor characteristics, such as tumor site, tumor invasion stage (T), and nodal stage (N) (**Supplementary Table 1**). A total of 30 mutated genes were identified. There was no genetic variant significantly associated with recurrence in these CRC patients by odds ratio. The hazard ratio (**Supplementary Figure 1B**) of four mutated genes, including *MTOR*, *BAP1*, *TSC1*, and *NOTCH1*, showed a correlation with worse progression-free survival [p < 0.05 and hazard ratio (HR) =10.5–76.5]. However, these four genetic variants were rare and were found only in 1.3% (1/78) of these CRC patients (**Supplementary Figure 1C**). Targeting these rare mutations does not seem to provide significant improvements in the clinical outcome of stage III CRC. These data imply the limitation of the conventional approach of the analytic sequencing method.

### Targeting Intratumor Heterogeneity by Cancer Evolution Model

**Figure 1:**
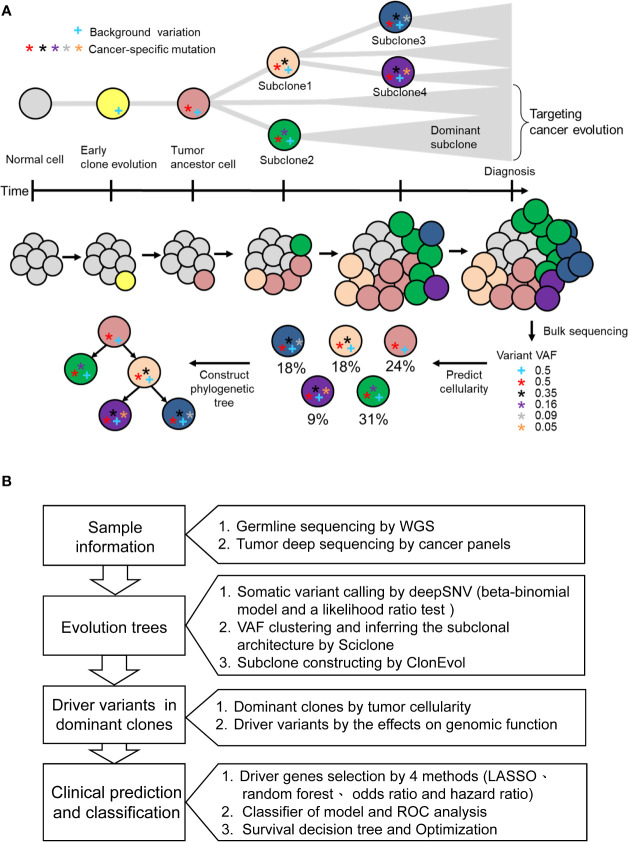
The concept of cancer evolution and study design. **(A)** The cancer evolution model depicts the accumulation of cancer-specific mutations and clonal expansion during tumor formation. The data of VAF generated from deep sequencing of tumor tissues could be used to predict the cellularity and construct the phylogenetic tree. The blue cross indicates the background variation. Stars with different colors represent mutations that develop during cancer evolution. **(B)** Based on the concept of cancer evolution, the research design is proposed to identify driver mutations with crucial clinical impact.

Evolutionary dynamic models have been studied to elucidate the process of tumorigenesis and discover the driver somatic mutations for the development of potential therapeutic strategies (25). Accordingly, we built clonal evolution models and applied statistics and machine learning algorithms to identify disease-related driver mutations. As shown in **Figure 1A**, normal cells accumulate background variations (blue cross) and many cancer-specific mutations (stars) over a prolonged clinically latent period to become cancers. Multiple subclones could be found within single cancer tissue. Theoretically, the background variation and driver mutation (red star) are present in the ancestor cell and all the subclones. During cancer evolution, additional mutations occur subsequently in different subclones, which give rise to intratumoral heterogeneity. Deep targeted-gene sequencing of bulky tumor tissue provides useful information on variant allele frequency (VAF), which could be used to predict cellularity and construct phylogenetic trees. Therefore, we used the targeted-gene sequencing data of 78 stage III CRCs to reconstruct the tumor evolution. The WGS data from paired normal blood samples were used to filter germline variants (**Figure 1B**). After the somatic variant calling by DeepSNV (13), SciClone (14) was applied for analyzing the distribution of purity-scaled variant allele fractions, and ClonEvol (15) was used to reconstruct the phylogenetic tree. We determined the dominant clone according to the predicted cellularity. After that, the potential candidate driver mutations in ancestor and dominant clones could be identified. We applied machine learning models to predict the risk of cancer recurrence by using different genetic variant feature selection strategies and classifiers. By this pipeline, we could identify the critical driver genetic variants that could be potential drug targeting clonal variants and involved in cancer survival stratification (**Figure 1B**).

### Phylogenetic Tree Reconstruction From the Clonal Evolution Model

**Figure 2:**
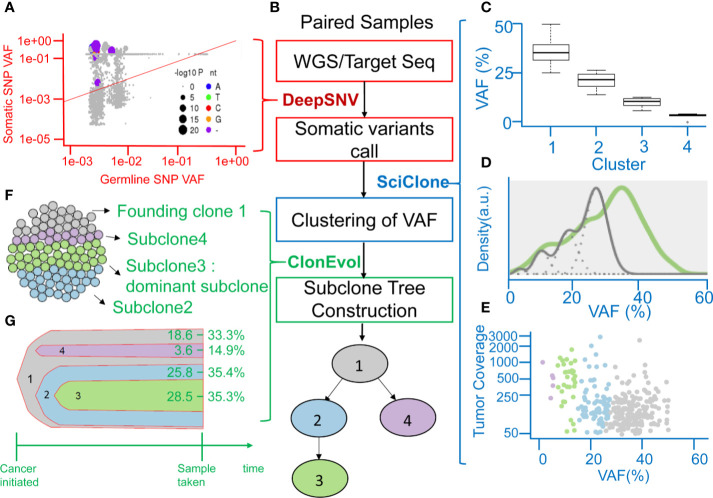
Representation of analyzing clonal evolution in a CRC patient as an example. **(A)** Scatter plot showing the variant allele frequencies (VAFs) of the example *VHL* gene in tumor and normal tissues and the levels of significance of the deepSNV test. **(B)** The flow chart showing the utility of various algorithms for calling of SNV, clustering of VAFs, and analysis of clonal relationships from the sequencing data of paired normal-tumor samples. **(C)** Inferring subclones according to the clustering of VAFs by SciClone. **(D)** Kernel density estimation (KDE) of VAFs. The distribution of mutations occurring in colon cancer patients. The gray line represents the mutations detected in the founding clone, and the green line represents driver mutations in the dominant subclone. **(E)** The read depth versus VAF plot showing the read depth of 4 clusters of genes under the assumption of neutral copy number (copy number = 2). **(F)** Sphere of cells demonstrating the founding clone and clonal subpopulations of the CRC tumor sample by CloneEvol. **(G)** Bell plot showing clonal dynamics during tumor formation.

A clinical example is shown in **Figure 2**. This case was a 40-year-old man with stage III CRC at initial diagnosis. He received standard surgical resection followed by adjuvant chemotherapy with mFOLFOX6. Recurrence was detected by computed tomography (CT) scan 15.4 months after surgery. WGS and deep targeted-gene sequencing were performed on paired normal and tumor samples, respectively. **Figure 2A** displays the allele frequency of the detected variants in tumor and germline tissues and indicates the levels of significance of the deepSNV test. Using the *VHL* gene as an example, the dots above the diagonal line represented the variants that were called as true variants rather than sequencing errors by the deepSNV algorithm. A total of 307 somatic SNVs were detected in this case. When SciClone was used to perform the clustering, 307 SNVs clustered into four groups (**Figure 2C**). The mean VAF values of clusters 1 to 4 are 36.5, 22.1, 11.2, and 4.2%. Besides, the mean posterior probabilities of clusters 1 to 4 are 93.5, 83, 95, and 99%. **Figure 2D** shows the kernel density plots of VAF under two copy number estimations. The model did not perfectly fit the original distribution because the mean probability of cluster 2 was 83%. **Figure 2E** shows the scatter plot of each cluster’s SNV coverage and VAF. The SNVs with coverage less than 50x were filtered out in this study because the low coverage would lead to biased estimation. **Figures 2F, G** demonstrate the cancer cellularity prediction and the most likely evolutionary tree of the primary tumor *via* ClonEvol. In this case, the gray color was the founding clone with cellularity ranging from 18.6–33.3%, the green color was the dominant subclone ranging from 28.5–35.3%, the blue color was subclone two ranging from 25.8–35.4%, and the purple color was subclone four ranging from 3.6–14.9%. Finally, the phylogenetic tree was constructed (**Figure 2B**).

### Sequential Oncogenic and Tumor Suppression Genetic Alterations in Cancer Evolution

**Figure 3:**
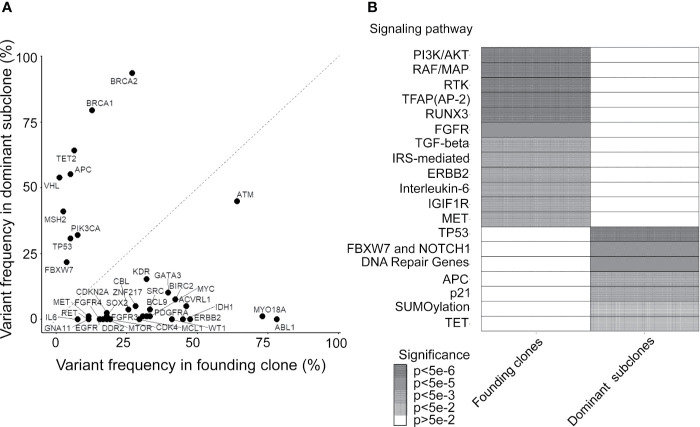
Mutated genes and relevant pathways in the founding clone and dominant subclones. **(A)** Percentage of patients who harbor each mutated gene was occurring in founding clones or dominant subclones of their primary tumors. A gene under the dashed diagonal indicates that mutation frequently occurs in the founding clone than the dominant subclone of these patients. **(B)** Heatmap showing oncogenic pathways enriched by mutated genes frequently occurring in founding clones and dominant subclones, respectively. Color density represents the significance of enrichment derived by Reactome.

By using the study protocol shown in **Figure 1B**, we identified possible driver mutations in the founding clone and dominant subclone for each cancer patient. Among 78 CRC patients, 66 and 49 genetic variants with high or moderate protein impact were detected in founding and dominant subclones, respectively. Several variants were frequently detected in the founding clones of these 78 CRCs, including the *ABL1*, *MYO18A*, and *ATM* mutations (**Figure 3A**). Approximately 78.2, 73.1, and 64.1% of patients harbored the *ABL1*, *MYO18A*, and *ATM* mutations, respectively, in their founding clone. In contrast, the most commonly detected mutations in the dominant subclones of these CRCs were *BRCA1*, *BRCA2*, *TET2*, *APC*, *VHL*, *MSH2*, *TP53*, *PIK3CA*, and *FBXW7* mutations, accounting for 79.5, 93.6, 64.1, 55.1, 53.8, 41, 30.8, 32, and 21.8%, respectively. Driver genes can be classified into distinct signaling pathways that control cell survival, cell fate, and genome maintenance (26). Accordingly, we analyzed the dysregulated signaling pathways in founding clones and dominant subclones. As shown in **Figure 3B**, the signaling pathways involved were significantly different between the founding clones and dominant subclones. Alterations in the *PI3K*/*AKT*, *RAF*/*MAP*, and RTK signaling pathways were commonly detected in founding clones. By contrast, the dominant subclones frequently exhibited dysregulations in the *TP53*, *FBXW7*/*NOTCH1*, and DNA repair pathways. These results implied that cancer cells accumulated different somatic mutations during cancer evolution. Oncogenic alterations in signaling pathways controlling cell proliferation and survival, such as the *PI3K*/*AKT* and *MAPK* pathways, occurred at the early stage of cancer formation (27, 28). Mutations involving the tumor suppressors and DNA repair pathways became more important during evolution.

### Optimizing the Selection of Clonal Mutations by Recurrence Status

**Figure 4:**
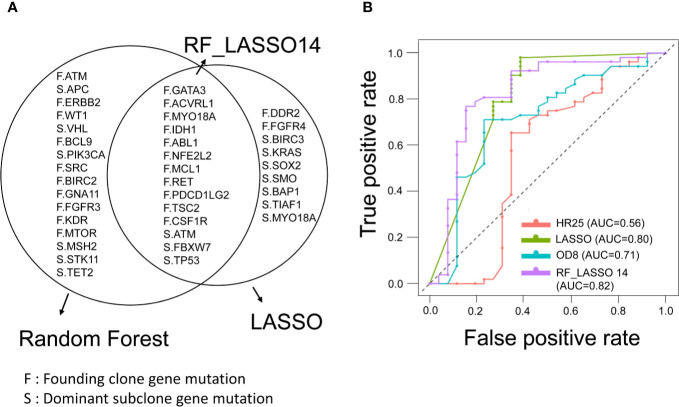
Feature selection of founding clone and dominant subclone genes and prediction model for CRC recurrence. **(A)** Venn diagram showing overlapping genes selected by the random forest (RF) and LASSO models. RF_LASSO14 indicates a group of 14 overlapping genes. F represents the founding clone gene mutation, and S represents the dominant subclone gene mutation. **(B)** Receiver operating characteristic (ROC) curves of gene sets selected by different statistical and machine learning models for prediction of recurrence in stage III CRCs. The area under the curve of ROC corresponding to each gene set was shown. RF_LASSO14, 14 genes simultaneously selected by random forest and LASSO; OD8, eight genes selected by odds ratio; HR25, 25 genes selected by hazard ratio.

To investigate the clinical significance of mutations detected in the founding clone and dominant subclones, we used different feature selection techniques to identify the important mutations associated with the recurrence of CRC patients. Top 30 mutations were selected by Gini importance using the random forest (RF). The *FBXW7* and *MYO18A* mutations were the variables with the highest importance among these CRC patients. LASSO was performed to select mutated genes with nonzero coefficients, and 23 mutations (LASSO23) were selected. By calculating the odds ratio (OD) and hazard ratio (HR), we identified 8 (OD8) and 25 (HR25) genes, respectively, that were significantly associated with recurrence in this CRC cohort (p < 0.05). The above results were shown in **Supplementary Table 2**. Fourteen genes were simultaneously selected by random forest and LASSO, including *GATA3*, *ACVRL1*, *MYO18A*, *IDH1*, *ABL1*, *NFE2L2*, *MCL1*, *RET*, *PDCD1LG2*, *TSC2*, *CSF1R*, *ATM*, *FBXW7*, and *TP53*. RF_LASSO14 was named for this 14-gene set (**Figure 4A**). We analyzed the correlation between disease-free survival (DFS) and the mutation status of 14 genes selected by both the random forest and lasso. Among these 14 genes, mutations of *CSF1R*, *PDCD1LG2*, *FBXW7*, *TSC2*, and *NFE2L2* gene were significantly associated with shorter DFS. In contrast, *MYO18A* mutation was associated with better DFS. No association between the DFS and the mutation status of the other eight genes was observed (**Supplementary Table 3**). The heatmap of these 14 mutated genes is shown in **Supplementary Figure 2**. For robust optimization, we input the gene groups identified by four different feature sets into five classifier models, including support vector machine (SVM), C5.0, random forest, logistic regression, and XGBoost (gradient boosting), to predict and classify the cancer recurrence. The recall value of 10-fold cross-validation for SVM, C5.0, RF, LR, and XGBoost were 0.615, 0.346, 0.384, 0.654, and 0.461, respectively. The accuracy for SVM, C5.0, RF, LR, and XGBoost were 0.756, 0.615, 0.756, 0.795, and 0.744, respectively. The logistic regression model had the best performance with the highest recall value and better accuracy. The results were shown in **Supplementary Figure 3**. The receiver operating characteristic (ROC) curve analysis confirmed that the RF_LASSO14 gene set had better accuracy (79%), precision (70%), and recall (65%) and area under the curve (AUC) (82%) in the logistic regression model (**Figure 4B**). To emphasize the importance of cancer evolution, we compared the performance between the models with or without intratumor heterogeneity. We operated the bootstrapping process 5000 times and selected background genetic mutations as features by lasso methods and built the same logistic regression classifier to calculate the probability of accuracy over 0.795. Consequently, the probability of a conventional model with better accuracy than the evolution model was only 4.54%, which proved that our model was quite meaningful.

### Survival Stratification by Three Genetic Variants *via* the Decision Tree Model

**Figure 5:**
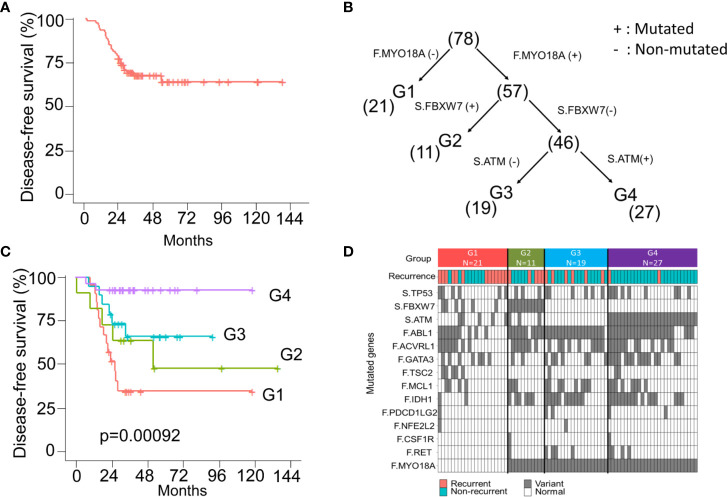
The model of survival decision tree (CRC) **(A)** The Kaplan-Meier curve of disease-free survival (DFS) for 78 stage III CRC patients. **(B)** CRC patients are grouped into four subgroups according to the mutation status of the *MYO18A* gene in the founding clone (F) MYO18A) and *FBXW7* and *ATM* genes in the dominant subclone (S. FBXW7 and S.ATM). Parentheses indicate the number of patients in each group. **(C)** The Kaplan-Meier curves of DFS stratified by group (1–4) and compared with the log-rank test. **(D)** Summary of the mutations and clinical outcome in 4 subgroups of CRC patients. The colored rectangles indicate the detection of recurrent disease or not. The gray and white rectangles indicate the gene with and without the mutation.

Currently, the “one size fits all” approach is still used for adjuvant treatment of stage III CRC patients. FOLFOX6 chemotherapy is the gold standard regimen without considering genomic alterations. In this CRC cohort, all patients were pathological stage III and received standard surgical resection followed by adjuvant FOLFOX6 chemotherapy. The 5-year DFS was approximately 70% (**Figure 5A**). The decision tree, a nonparametric supervised learning method, was used to analyze the predictive value of the mutations identified in the founding clone and dominant to further subclassify these stage III CRC patients and identify potential treatment strategies subclone. As shown in **Figure 5B**, three mutations, including the *MYO18A* mutation in the founding clone and *FBXW7* and the *ATM* mutation in the dominant subclone, could be used to stratify these 78 CRC patients into four subgroups that had different clinical outcomes. Group 1 (G1) was the patient without the *MYO18A* mutation in the founding clone (F.MYO18A), and this group of patients had the worst DFS (**Figure 5C**). Patients harboring the *MYO18A* mutation could be further categorized into groups 2, 3, and 4 according to the dominant subclone’s mutation status of *FBXW7* and *ATM*. Patients in group 2 (G2) had *FBXW7* mutations (S.FBXW7), and patients in group 4 (G4) had *ATM* mutations in the dominant subclone (S.ATM). Patients in group 4 had the best outcome, followed by groups 3 and 2. The mutated genes in the founding and dominant subclones detected in these four subgroups of patients are shown in **Figure 5D**. Targeting these relevant mutations in founding and dominant subclones might provide benefits for stage III CRC patients, especially groups 1 and 2. Since *MYO18A*, *FBXW7*, and *ATM* mutations (**Supplementary Figure 5**) have a considerable impact on clinical outcomes, these mutations might be potential therapeutic targets. *MYO18A* mutation was detected in 50 and 84.6% of patients with and without recurrence. In contrast, the percentage of *FBXW7* mutations in patients with or without recurrence was 38.5 and 13.5%, respectively. When analyzed by the Chi-Square test, the distributions of *MYO18A* and *FBXW7* mutations are significantly different in these two groups of patients (p = 0.002 and 0.019) (**Supplementary Table 1**). Several clinical and pathological factors, as shown in the **Supplementary Table 4**, were also considered when analyzing the prognostic impact of *MYO18A* and *FBXW7* mutations. We analyzed these factors through univariate and multivariate Cox proportional hazards model. The results showed *MYO18A*, and *FBXW7* mutations are significantly associated with the clinical outcome when univariate analysis. The outcome was not affected by age, gender, primary tumor location, the depth of tumor invasion, and the number of lymph node metastasis. In multivariate analysis, both the *MYO18A* and *FBXW7* mutations were still the independent prognostic factors (**Supplementary Table 4**).

### Examining the Biological Role of MYO18A *In Vitro*

**Figure 6:**
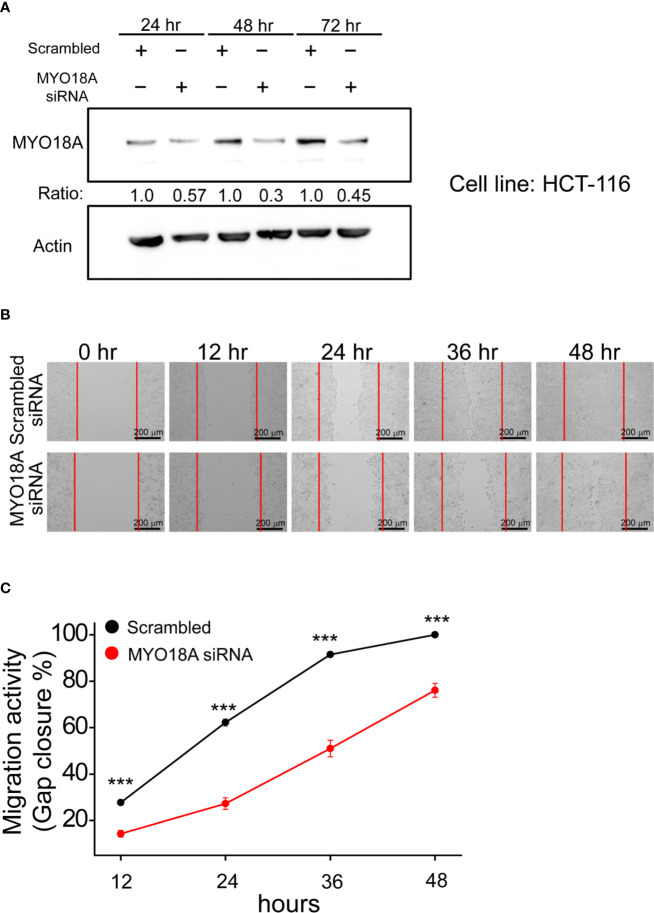
Depletion of MYO18A by siRNA suppressed cell migration in human CRC cells. **(A)** HCT-116 cells were transfected with either scrambled or *MYO18A* siRNA. Cells were harvested 24, 48, and 72 hours after transfection, and western blotting was used to determine the effect on MYO18A protein expression. Representative images **(B)** and quantitative analysis **(C)** of gap closure assay in HCT-116 cells. Forty-eight hours after the transfection of siRNA, the gap was created and monitored with a microscope every 12 hours. The red dotted lines indicate where the migration began. The total gap area created at 0 hours and gap closure areas at the indicated time points were quantified by ImageJ software. The migration activity, which was determined by the percentage of gap closure area, was compared between cells transfected with the scramble and *MYO18A* siRNA. Each value represents the mean +/- SEM from at least three independent experiments in each group. ***p < 0.01.

*MYO18A* is a gene encoding a unique myosin involved in intracellular transport processes and cell motilities (29). Therefore, we assessed whether *MYO18A* has a role in CRC cell invasion or migration. Specific siRNA targeting *MYO18A* was transfected into human CRC cancer cells, and the impact on cell migration was determined by gap closure assay. As shown in **Figure 6A**, *MYO18A* siRNA significantly reduced the level of *MYO18A* protein after 48 hours of transfection. Compared to cells treated with scrambled siRNA, HCT-116 cells transfected with *MYO18A* siRNA demonstrated a significant reduction in cell migration activity by 20 to 40% (**Figures 6B, C**). Reduced migration was also observed in *MYO18A* siRNA-treated HT-29 and DLD-1 cells. These results indicate that *MYO18A* plays an essential role in the migration of human CRC cells.

## Discussion

With the advances of NGS technologies and machine learning in cancer biology, targeting cancer evolution has become more feasible. Here, we demonstrate using a genomic-machine learning model for recurrence-risk prediction and identification of potential therapeutic targets for CRC. Importantly, we designed different treatment strategies for different risk subgroups of CRC patients. Our results highlight the following important points: (i) The sequential oncogenic and tumor suppression genetic alterations were found during tumor evolution. (ii) We identified a fourteen genes panel that could predict the risk of recurrence in stage III CRC. (iii) Three genes, including *MYO18A* in the founding clone, *FBXW7*, and *ATM* in the dominant subclone, affected the prognosis. (iv) MYO18A plays an important role in the migration of human CRC cells. These findings suggest that the integration of genomic data and cancer evolution models provides insights into disease biology. These results could be applied for the recurrence-risk classification of stage III CRC and the development of novel therapeutic strategies.

The ability to predict the future behavior of individual cancers is crucial for precision cancer medicine. Considering that traditional methods might hinder the efficacy of rare somatic selection, we established a more comprehensive pipeline by the cancer evolution model for treatment strategy analysis. First, we selected rare somatic mutations that were not detected by traditional methods. To target intratumor heterogeneity and cancer evolution somatic mutations to overcome chemotherapy resistance, we used the evolution model. We supposed that founding clone and dominant clones (the most estimated prevalence) are the significant events for cancer recurrence, which was confirmed by the probability of random two subclone sampling (**Supplementary Figure 5**). Finally, for robust optimization, the different machine learning algorithms and statistical methods were selected for cancer recurrence-risk prediction and survival stratification models.

In the CRC multistage progression model, the adenoma-carcinoma sequence refers to a stepwise pattern of mutational activation of oncogenes and inactivation of tumor suppressor genes. In our cancer evolution model, we provide information about genetic changes in cancer-driving metastasis. In the early stages, mutations in the oncogenic pathway, such as the receptor kinase signaling (RTK) pathway, the fibroblast growth factor receptor (FGFR) signaling pathway, and the transforming growth factor-beta (TGFB) signaling pathway, appear to be the first step. Second, mutations in *TP53*, *FBXW7*, and *APC* may play a role in cancer evolution. Sequential oncogenic and tumor suppression genetic alterations were consistent with the hypothesis of cancer two-hit theory.

Classification and decision systems in data analysis are mostly based on accuracy. In our study, we trade off accuracy, precision, and recall for useful optimization in a multiple machine learning model. We selected the 14 genetic variants for cancer recurrence prediction. The variant distribution and frequency in cancer patients with or without recurrence are shown in **Figure 4**. There are 11 genetic variants in the funding clone and three genetic variants in the dominant subclone. This study implies a robust optimization cancer panel for recurrence prediction. We have developed a genomic-machine learning model and pipeline software for CRC recurrence-risk prediction.

**Figure 5A** shows the 5-year DFS of this CRC cohort. The 5-year disease-free survival rate is approximately 70%, which has reached the benchmark of a worldwide standard. In addition to modeling for recurrence prediction, we need to improve care survival by different treatment strategies. Using the three machine learning models, we can classify the CRC subgroup by three genetic variants. Group 1 and group 2 have a poor prognosis. The progression-free survival of Group 4 was better than that of group 3. This successful study identified associations between three genetic markers and survival subgroup and recurrence status. The uniqueness of this study is that the evolution model shows the clinical impact on stage III colorectal cancer by the machine learning method utilizing the comprehensive clinical and genomic information. However, the major limitation of this study is the small sample size. It is too early to make a strong conclusion at this stage in terms of the case number.

*MYO18A* and *FBXW7* intratumor heterogeneity variants are potential targets in the cancer evolution model. *MYO18A* is an unconventional myosin that has been implicated in multiple cellular processes. *MYO18A* has been involved as a cancer driver. Overexpression of *MYO18A* was observed in metastatic prostate cancer cell lines in a previous study (29, 30). Migration assay of various cancer cell lines also revealed that *MYO18A*-depleted cells had decreased cell motility (31, 32). By analyzing the clinical data, we found that patients without *MYO18A* mutation in the founding clone (F. MYO18A) had the worst DFS (**Figures 5B, C**). Moreover, the *in vitro* study showed knockdown of *MY018A* by siRNA caused a reduction in cell migration activity by 20–40% (**Figure 6**). These results imply that *MYO18A* is a potential tumor driver for cancer cell migration. The alteration of *MYO18A* was common in the founding clone (**Figure 3**). To conclude, the alteration of *MY018A* is a gain-of-function or loss-of-function mutation; further *in vitro* and *in vivo* investigations are needed to study the impact of *MYO18A* mutations on cell survival, proliferation, or angiogenesis. *FBXW7* is a critical tumor suppressor involved in the ubiquitin-proteasome system in human cancer. It has been demonstrated that metastatic CRC patients with *FBXW7* missense mutations show shorter overall survival compared with patients with wild-type *FBXW7* (33). The results are consistent with our data showing poor prognosis survival in the G1 and G2 groups.

In conclusion, this study highlights the importance of a cancer evolution model in the development of new therapeutic strategies. The integration of genomics and machine learning could provide an opportunity to identify new targets for cancers.

## Data Availability Statement

The datasets presented in this study can be found in online repositories. The names of the repository/repositories and accession number(s) can be found below: NCBI BioProject (Accessions: PRJNA655796/PRJNA662159).

## Ethics Statement

This study was approved by the institutional review board of NCKUH (A-ER-103-395 and A-ER-104-153) and conducted under the Declaration of Helsinki. All participants provided written informed consent.

## Author Contributions

Conception and study design: P-CL and M-RS. Development of methodology: P-CL, Y-MY, and M-RS. Acquisition of data: Y-MY, B-WL, S-CL, and P-CC. Statistical and computational analysis: P-CL, Y-MY, and M-RS. Writing, review, and/or revision of the manuscript: P-CL, Y-MY, B-WL, S-CL, R-HC, P-CC, and M-RS. Study supervision: M-RS. All authors contributed to the article and approved the submitted version.

## Funding

This work was supported by the Ministry of Science and Technology (MOST 104-2320-B-006-015-MY3, MOST 107-2319-B-006-001 and MOST 108-2319-B-006-001 to M-RS), National Health Research Institutes (NHRI-108A1-CACO-02181811), the Ministry of Health Welfare (MOHW107-TDU-B-211-114018, MOHW108-TDU-B-211-124018), National Cheng Kung University and National Cheng Kung University Hospital, Taiwan.

## Conflict of Interest

The authors declare that the research was conducted in the absence of any commercial or financial relationships that could be construed as a potential conflict of interest.
