# Supplementary material for: Intratumor Heterogeneity of MYO18A and FBXW7 Variants Impact the Clinical Outcome of Stage III Colorectal Cancer
Source: Front Oncol. 2020 Oct 29;10:588557. doi: 10.3389/fonc.2020.588557 (PMC7658598; doi:10.3389/fonc.2020.588557)
Supplement: Supplementary file 1 [file Presentation_1.pptx]

## Slide 1
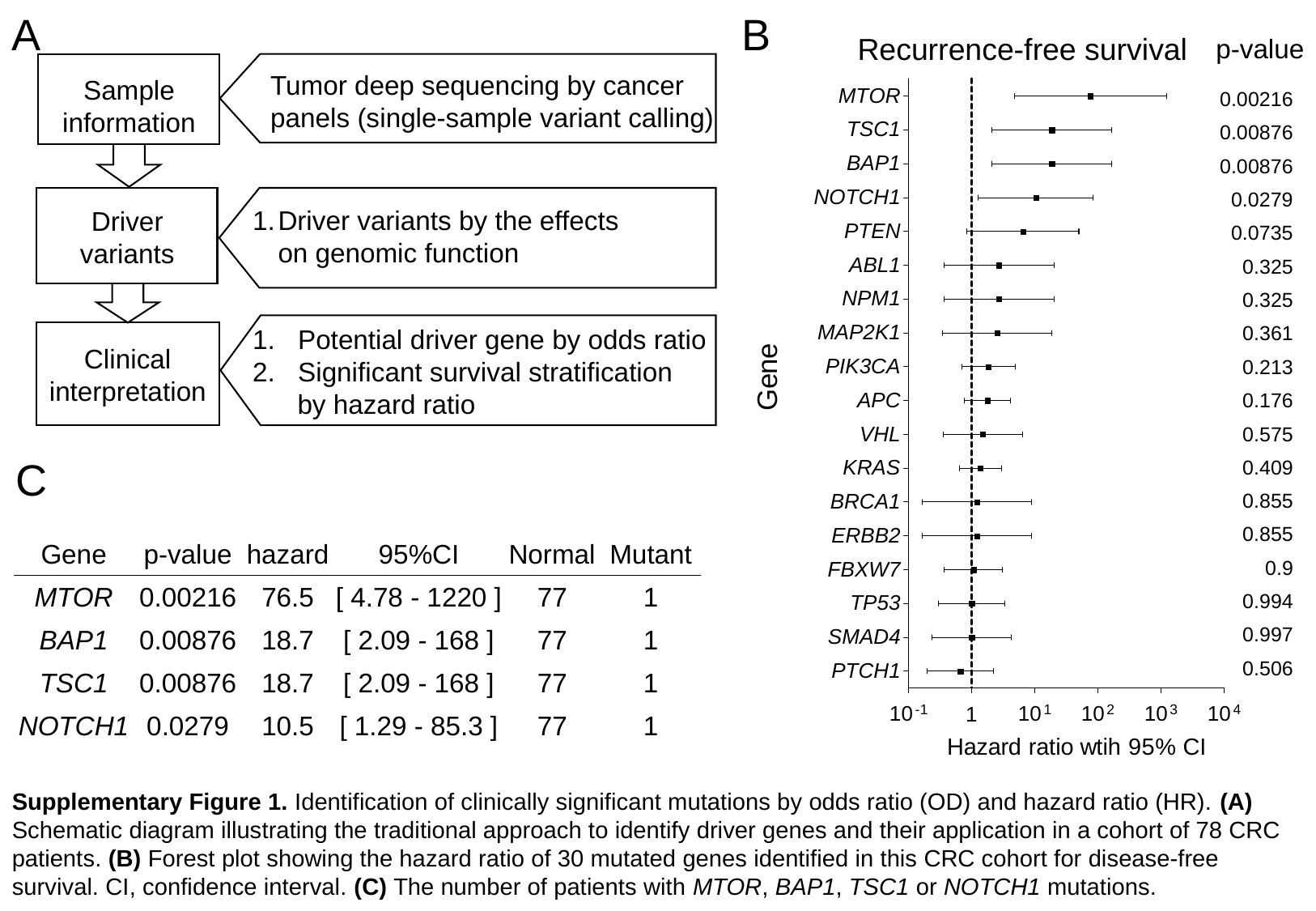

A
B
Recurrence-free survival
p-value
Tumor deep sequencing by cancer
panels (single-sample variant calling)
Sample information
Driver variants
Driver variants by the effects on genomic function
 Clinical
interpretation
| 0.00216 |
| --- |
| 0.00876 |
| 0.00876 |
| 0.0279 |
| 0.0735 |
| 0.325 |
| 0.325 |
| 0.361 |
| 0.213 |
| 0.176 |
| 0.575 |
| 0.409 |
| 0.855 |
| 0.855 |
| 0.9 |
| 0.994 |
| 0.997 |
| 0.506 |
Potential driver gene by odds ratio
Significant survival stratification
 by hazard ratio
C
| Gene | p-value | hazard | 95%CI | Normal | Mutant |
| --- | --- | --- | --- | --- | --- |
| MTOR | 0.00216 | 76.5 | [ 4.78 - 1220 ] | 77 | 1 |
| BAP1 | 0.00876 | 18.7 | [ 2.09 - 168 ] | 77 | 1 |
| TSC1 | 0.00876 | 18.7 | [ 2.09 - 168 ] | 77 | 1 |
| NOTCH1 | 0.0279 | 10.5 | [ 1.29 - 85.3 ] | 77 | 1 |
Supplementary Figure 1. Identification of clinically significant mutations by odds ratio (OD) and hazard ratio (HR). (A) Schematic diagram illustrating the traditional approach to identify driver genes and their application in a cohort of 78 CRC patients. (B) Forest plot showing the hazard ratio of 30 mutated genes identified in this CRC cohort for disease-free survival. CI, confidence interval. (C) The number of patients with MTOR, BAP1, TSC1 or NOTCH1 mutations.
