# Supplementary material for: Intratumor Heterogeneity of MYO18A and FBXW7 Variants Impact the Clinical Outcome of Stage III Colorectal Cancer
Source: Front Oncol. 2020 Oct 29;10:588557. doi: 10.3389/fonc.2020.588557 (PMC7658598; doi:10.3389/fonc.2020.588557)
Supplement: Supplementary file 2 [file Presentation_2.pptx]

## Slide 1
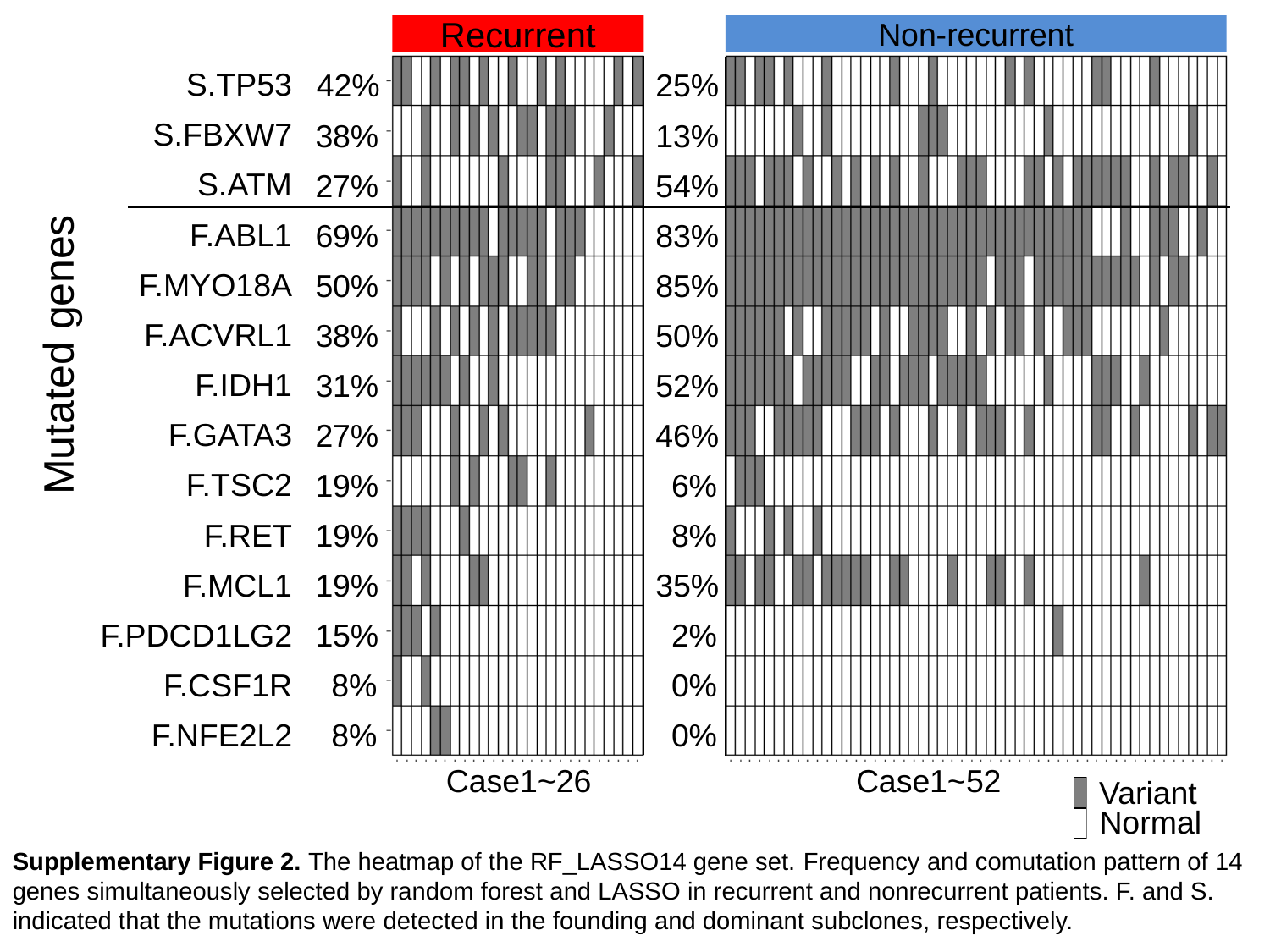

Recurrent
Non-recurrent
S.TP53
42%
25%
S.FBXW7
38%
13%
S.ATM
27%
54%
F.ABL1
69%
83%
F.MYO18A
50%
85%
F.ACVRL1
38%
50%
Mutated genes
F.IDH1
31%
52%
F.GATA3
27%
46%
F.TSC2
19%
6%
F.RET
19%
8%
F.MCL1
19%
35%
F.PDCD1LG2
15%
2%
F.CSF1R
8%
0%
8%
0%
F.NFE2L2
Case1~26
Case1~52
Variant
Normal
Supplementary Figure 2. The heatmap of the RF_LASSO14 gene set. Frequency and comutation pattern of 14 genes simultaneously selected by random forest and LASSO in recurrent and nonrecurrent patients. F. and S. indicated that the mutations were detected in the founding and dominant subclones, respectively.
