# Supplementary material for: Intratumor Heterogeneity of MYO18A and FBXW7 Variants Impact the Clinical Outcome of Stage III Colorectal Cancer
Source: Front Oncol. 2020 Oct 29;10:588557. doi: 10.3389/fonc.2020.588557 (PMC7658598; doi:10.3389/fonc.2020.588557)
Supplement: Supplementary file 3 [file Presentation_3.pptx]

## Slide 1
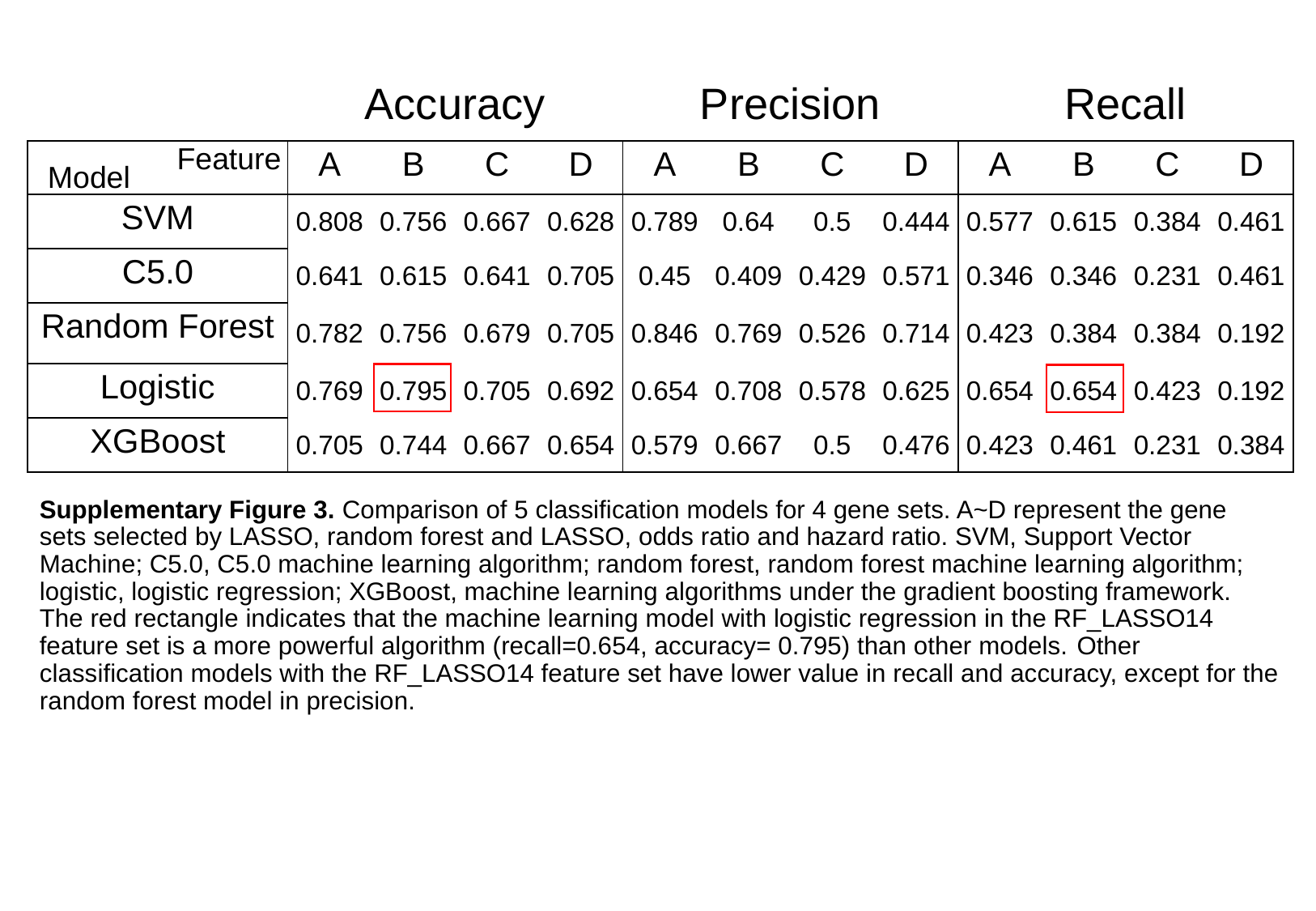

| | Accuracy | | | | Precision | | | | Recall | | | |
| --- | --- | --- | --- | --- | --- | --- | --- | --- | --- | --- | --- | --- |
| | A | B | C | D | A | B | C | D | A | B | C | D |
| SVM | 0.808 | 0.756 | 0.667 | 0.628 | 0.789 | 0.64 | 0.5 | 0.444 | 0.577 | 0.615 | 0.384 | 0.461 |
| C5.0 | 0.641 | 0.615 | 0.641 | 0.705 | 0.45 | 0.409 | 0.429 | 0.571 | 0.346 | 0.346 | 0.231 | 0.461 |
| Random Forest | 0.782 | 0.756 | 0.679 | 0.705 | 0.846 | 0.769 | 0.526 | 0.714 | 0.423 | 0.384 | 0.384 | 0.192 |
| Logistic | 0.769 | 0.795 | 0.705 | 0.692 | 0.654 | 0.708 | 0.578 | 0.625 | 0.654 | 0.654 | 0.423 | 0.192 |
| XGBoost | 0.705 | 0.744 | 0.667 | 0.654 | 0.579 | 0.667 | 0.5 | 0.476 | 0.423 | 0.461 | 0.231 | 0.384 |
Feature
Model
Supplementary Figure 3. Comparison of 5 classification models for 4 gene sets. A~D represent the gene sets selected by LASSO, random forest and LASSO, odds ratio and hazard ratio. SVM, Support Vector Machine; C5.0, C5.0 machine learning algorithm; random forest, random forest machine learning algorithm; logistic, logistic regression; XGBoost, machine learning algorithms under the gradient boosting framework. The red rectangle indicates that the machine learning model with logistic regression in the RF_LASSO14 feature set is a more powerful algorithm (recall=0.654, accuracy= 0.795) than other models. Other classification models with the RF_LASSO14 feature set have lower value in recall and accuracy, except for the random forest model in precision.
