# Supplementary material for: Intratumor Heterogeneity of MYO18A and FBXW7 Variants Impact the Clinical Outcome of Stage III Colorectal Cancer
Source: Front Oncol. 2020 Oct 29;10:588557. doi: 10.3389/fonc.2020.588557 (PMC7658598; doi:10.3389/fonc.2020.588557)
Supplement: Supplementary file 4 [file Presentation_4.pptx]

## Slide 1
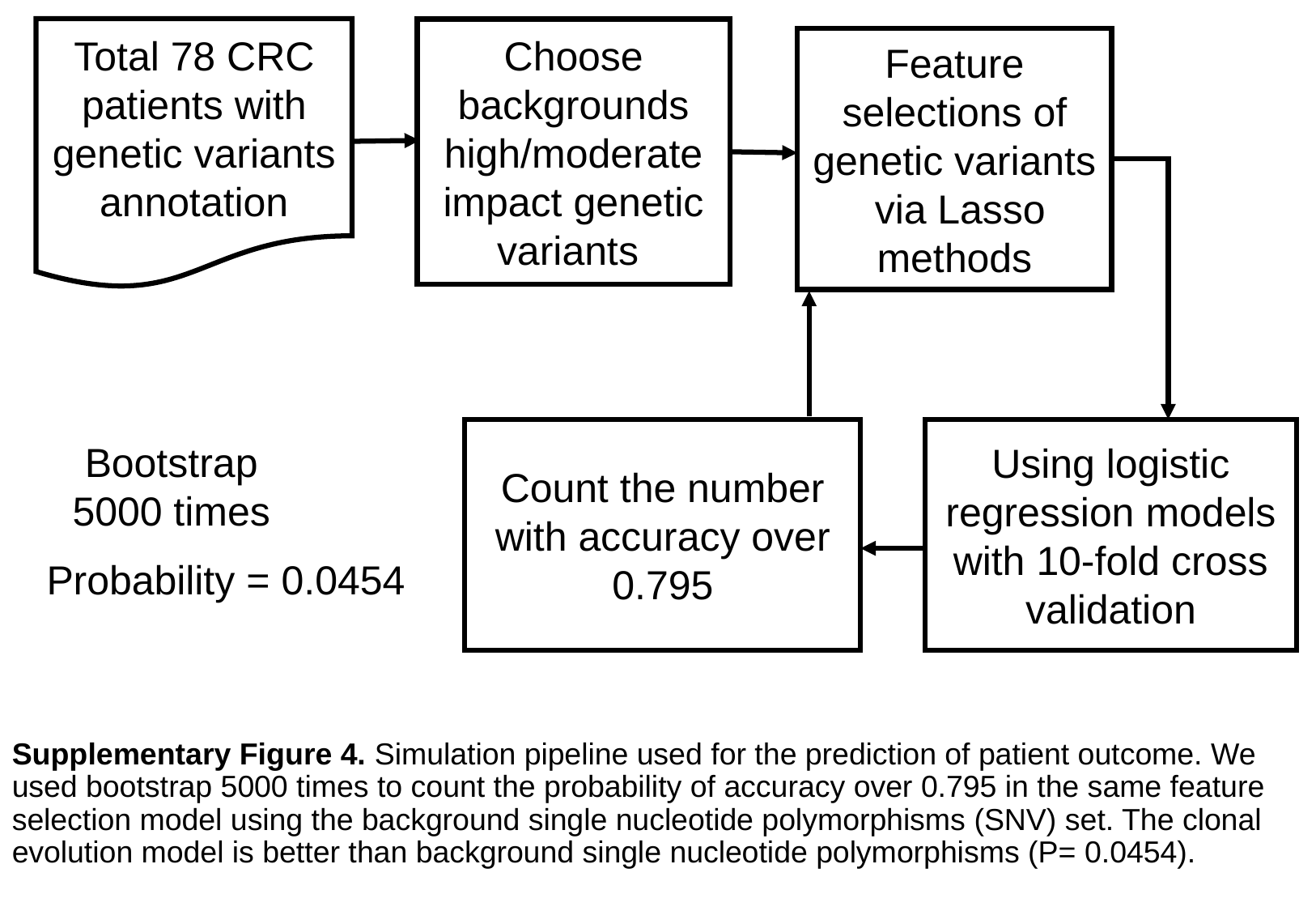

Total 78 CRC patients with genetic variants annotation
Choose backgrounds high/moderate impact genetic variants
Feature selections of genetic variants via Lasso methods
Count the number with accuracy over 0.795
Using logistic regression models with 10-fold cross validation
Bootstrap 5000 times
Probability = 0.0454
Supplementary Figure 4. Simulation pipeline used for the prediction of patient outcome. We used bootstrap 5000 times to count the probability of accuracy over 0.795 in the same feature selection model using the background single nucleotide polymorphisms (SNV) set. The clonal evolution model is better than background single nucleotide polymorphisms (P= 0.0454).
