# Supplementary material for: Intratumor Heterogeneity of MYO18A and FBXW7 Variants Impact the Clinical Outcome of Stage III Colorectal Cancer
Source: Front Oncol. 2020 Oct 29;10:588557. doi: 10.3389/fonc.2020.588557 (PMC7658598; doi:10.3389/fonc.2020.588557)
Supplement: Supplementary file 5 [file Presentation_5.pptx]

## Slide 1
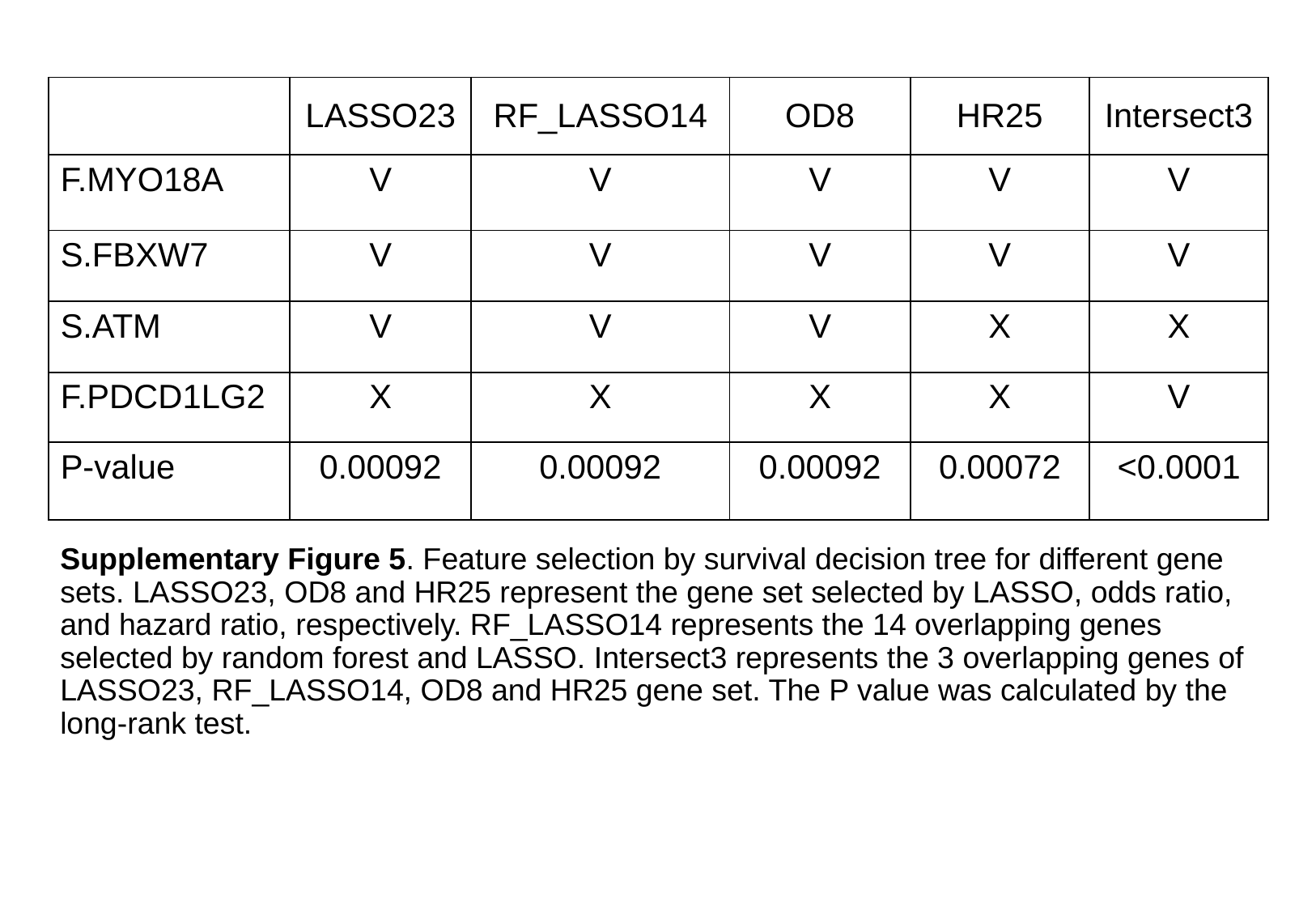

| | LASSO23 | RF\_LASSO14 | OD8 | HR25 | Intersect3 |
| --- | --- | --- | --- | --- | --- |
| F.MYO18A | V | V | V | V | V |
| S.FBXW7 | V | V | V | V | V |
| S.ATM | V | V | V | X | X |
| F.PDCD1LG2 | X | X | X | X | V |
| P-value | 0.00092 | 0.00092 | 0.00092 | 0.00072 | <0.0001 |
Supplementary Figure 5. Feature selection by survival decision tree for different gene sets. LASSO23, OD8 and HR25 represent the gene set selected by LASSO, odds ratio, and hazard ratio, respectively. RF_LASSO14 represents the 14 overlapping genes selected by random forest and LASSO. Intersect3 represents the 3 overlapping genes of LASSO23, RF_LASSO14, OD8 and HR25 gene set. The P value was calculated by the long-rank test.
