# Supplementary material for: Intratumor Heterogeneity of MYO18A and FBXW7 Variants Impact the Clinical Outcome of Stage III Colorectal Cancer
Source: Front Oncol. 2020 Oct 29;10:588557. doi: 10.3389/fonc.2020.588557 (PMC7658598; doi:10.3389/fonc.2020.588557)
Supplement: Supplementary file 6 [file Table_1.docx]

Supplementary table 1. Patient characteristic

| Characteristic | Recurrence  (N=26) | Non-recurrence  (N=52) | P value |
| --- | --- | --- | --- |
| Age |  |  | 0.429 |
| <65 | 17 (65.4%) | 39 (75%) |  |
| ≥65 | 9 (34.6%) | 13 (25%) |  |
| Gender |  |  | 0.811 |
| Male | 12 (46.2%) | 27 (51.9%) |  |
| Female | 14 (53.8%) | 25 (48.1%) |  |
| Tumor location |  |  | >0.99 |
| Left | 22 (84.6%) | 41 (78.8%) |  |
| Right | 4 (15.4%) | 9 (17.3%) |  |
| Tumor invasion |  |  | >0.99 |
| T1/T2 | 4 (15.4%) | 8 (15.4%) |  |
| T3/T4 | 22 (84.6%) | 44 (84.6%) |  |
| Lymph nodes |  |  | 0.6 |
| N0/N1 | 17 (65.4%) | 38 (73.1%) |  |
| N2  F.MYO18A | 9 (34.6%) | 14 (26.9%) | 0.002 |
| Wild type  Mutated | 13 (50%)  13 (50%) | 8 (15.4%)  44 (84.6%) |  |
| S.FBXW7  Wild type  Mutated | 16 (61.5%)  10 (38.5%) | 45 (86.5%)  7 (13.5%) | 0.019 |
