# Supplementary material for: Intratumor Heterogeneity of MYO18A and FBXW7 Variants Impact the Clinical Outcome of Stage III Colorectal Cancer
Source: Front Oncol. 2020 Oct 29;10:588557. doi: 10.3389/fonc.2020.588557 (PMC7658598; doi:10.3389/fonc.2020.588557)
Supplement: Supplementary file 9 [file Table_4.docx]

Supplementary table 4. Univariate and multivariate analysis for disease-free survival

|  | | Univariate analysis | | Multivariable analysis | |
| --- | --- | --- | --- | --- | --- |
| Characteristic | | HR(95%CI) | P value | HR(95%CI) | P value |
| Age | >=65 vs <65 | 1.6 (0.7-3.57) | 0.27 | 1.81 (0.67-4.92) | 0.24 |
| Gender | Male vs Female | 0.88 (0.41-1.9) | 0.74 | 0.88 (0.35-2.2) | 0.79 |
| Tumor location | Right vs Left | 0.97 (0.33-2.82) | 0.96 | 0.72 (0.23-2.34) | 0.59 |
| Tumor invasion | T1/T2 vs T3/T4 | 0.94 (0.32-2.72) | 0.9 | 1.38 (0.41-4.63) | 0.59 |
| Lymph nodes | N0/N1 vs N2 | 0.69 (0.31-1.54) | 0.36 | 0.52 (0.22-1.21) | 0.13 |
| F.MYO18A | Mutated vs Wild | 0.27 (0.13-0.6) | 0.0012 | 0.25 (0.1-0.62) | **0.003** |
| S.FBXW7 | Mutated vs Wild | 2.8 (1.3~6.19) | 0.011 | 3.52 (1.35~9.15) | **0.0098** |
